# 4072 Optimizing ex-vivo perfusion in Vascularized Composite Allotransplantation using Hyperosmolar solution and Electric Stimulation: Preliminary Results

**DOI:** 10.1017/cts.2020.87

**Published:** 2020-07-29

**Authors:** Michael Jonczyk, Philipp Tratnig-Frankl

**Affiliations:** 1 Tufts University; 2 Division of Plastic and Reconstructive Surgery, MGH

## Abstract

OBJECTIVES/GOALS: Vascularized composite allotransplantation (VCA) restores devastating soft tissue injuries. However, graft viability may be compromised during ischemia time, thus preservation techniques continue to evolve. Here we summarize our preliminary findings from preservation techniques utilizing hyperosmolar extracellular solution (HES) and electric stimulation in a 6 hour ex-vivo perfusion model. METHODS/STUDY POPULATION: A published, MGH rodent hindlimb ex-vivo perfusion model was utilized for this project. Three baseline control elements were taken to compare our results including: a baseline muscle biopsy, harvested hindlimb preserved on ice(4°C) in static cold storage (SCS), and 6 hour perfusion (SNMP) were used to compare results of our four aims. The four aims are shown in Table 1. HES was composed of muscle media and the addition of mannitol until 3 concentrations were made: 300, 500, and 800 mOsm concentrations. In aim 4, the perfusate composition was changed to test a hyper-oncotic purfusate. After 6 hours a muscle biopsy was taken to analyze energy cofactors via liquid chromatography-mass spectrometry, referred to as energy charge. Weight gain (edema), lactate levels, oxygen consumption and energy charge (EC) were used as markers for muscle tissue viability. RESULTS/ANTICIPATED RESULTS: In Aim 1, the higher osmolarity of HES indirectly reduced weight gain but consequentially reduced the EC below 5% when compared to SCS control group. We next incorporate HES into the perfusion model, Aim 2, and noticed a diminution in weight gain. The 500 mOsm group had substantial improvement in EC, lactate production and improved oxygen exchange when compared to a controls: fresh muscle biopsy and SNMP. In Aim 3, after a 6 hour perfusion with the addition of electric stimulation, graft edema improved by 10%, EC improved by 23% and O2 dissociation was highest of all 4 aims. Consequentially, due to muscular contraction the lactate levels were highest. In Aim 4, using a hyper-oncotic perfusate, edema reduced the most during the 6 hour perfusion but revealed lower EC and similar lactate/O2 results. However when left on for 24 hours, edema was significantly higher with lactate build up, EC improved with time as well as O2 dissociation. DISCUSSION/SIGNIFICANCE OF IMPACT: Here we have shown our preliminary results comparing our known ex-vivo perfusion model to multiple hypothesis to improve VCA graft viability. These preservation techniques demonstrate promising results but further studies are ongoing to confirm this encouraging outcome.

