# 4072 Optimizing ex-vivo perfusion in Vascularized Composite Allotransplantation using Hyperosmolar solution and Electric Stimulation: Preliminary Results – ERRATUM

**DOI:** 10.1017/cts.2020.503

**Published:** 2023-02-28

**Authors:** Michael Jonczyk, Philipp Tratnig-Frankl, Korkut Uygun, Curtis L. Cetrulo

The above abstract [1] published without listing Korkut Uygun and Curtis L. Cetrulo as authors.

The original article has been corrected online to rectify this error.
